# Cytomegalovirus-Reactive IgG Correlates with Increased IL-6 and IL-1β Levels, Affecting Eating Behaviours and Tactile Sensitivity in Children with Autism

**DOI:** 10.3390/biomedicines13020338

**Published:** 2025-02-02

**Authors:** Isti Anindya, Rini Sekartini, Ibnu Agus Ariyanto, Tjhin Wiguna, Novika Purnama Sari, Yuni Sri Rahayu, Amin Soebandrio

**Affiliations:** 1 Doctoral Program in Biomedical Sciences, Faculty of Medicine, Universitas Indonesia, Jakarta 10430, Indonesia; istianindya@gmail.com; 2 Department of Pediatrics, Dr. Cipto Mangunkusumo Hospital, Faculty of Medicine, Universitas Indonesia, Jakarta 10430, Indonesia; rsekartini@yahoo.com; 3 Department of Clinical Microbiology, Dr. Cipto Mangunkusumo Hospital, Faculty of Medicine, Universitas Indonesia, Jakarta 10430, Indonesia; ibnu.agus71@ui.ac.id; 4 Department of Psychiatry, Dr. Cipto Mangunkusumo Hospital, Faculty of Medicine, Universitas Indonesia, Jakarta 10430, Indonesia; twiga00@yahoo.com; 5 Department Clinical & Developmental Neuropsychology, Faculty of Behavioural and Social Sciences, University of Groningen, 9712 TS Groningen, The Netherlands; n.purnama.sari@rug.nl; 6 Pharma Metric Labs, Jakarta 10520, Indonesia; rahayuyunisri@gmail.com

**Keywords:** autism, cytokine imbalance, cytomegalovirus, eating behaviours, sensory processing

## Abstract

Background/Objectives: Elevated cytokine levels, including IL-6 and IL-1β, can contribute to persistent brain inflammation in children with autism and cytomegalovirus (CMV) infection, exacerbating autism-related behaviours and symptoms. This study evaluates the impact of CMV-induced cytokine increases on the eating behaviours and sensory profiles of children with autism. Methods: A cross-sectional design was employed, involving children aged two to five years (CMV-reactive IgG), with ASD (*n*= 98) and TD (*n* = 96). Serological tests using ELISA were conducted to measure IgG CMV, IL-6, and IL-1β biomarkers. Eating behaviours were evaluated using the BAMBI (Brief Autism Mealtime Behaviour Inventory), and sensory profiles were assessed using the SSP (Short Sensory Profile). Statistical analyses were performed using Spearman’s rank and chi-square tests. Results: The results show that autism significantly affects children’s eating behaviours and sensory profiles (*p* < 0.001), with notable differences found between the groups. Correlation analysis revealed a significant association between IgG CMV and IL-6 (*p* = 0.026) and IL-1β (*p* = 0.014) in the ASD group. Additionally, eating behaviours (food refusal and limited variety) in ASD correlated with IL-6 and IL-1β. Sensory characteristics, such as tactile sensitivity, were found to correlate with IL-6 (*p* = 0.027) and IL-1β (*p* = 0.002) in the ASD group. Conclusions: These findings suggest that CMV-infected children with autism are at increased risk of IL-6 and IL-1β dysregulation, contributing to sensory processing issues and eating behaviours. Further research is needed to enhance CMV testing protocols and better understand the virus’s role in the development of sensory and behavioural issues in children with autism.

## 1. Introduction

The prevalence of cytomegalovirus (CMV) varies globally and is influenced by geographic, socioeconomic, and medical factors that affect the infection’s distribution [1]. Globally, CMV seroprevalence among women of reproductive age is approximately 86%, with the Eastern Mediterranean region showing the highest rate at 92% [2]. Among children aged 1–5 years, CMV seroprevalence increased from 20.7% in 2011–2012 to 29.0% in 2017–2020, as reported by the National Health and Nutrition Examination Survey (NHANES) [3]. In Italy, a 2023 report found that 70–80% of women of reproductive age were CMV-seropositive [4].

The prevalence of CMV in Southeast Asia is inadequately documented, especially in Indonesia [5]. A study conducted in Jakarta found that 5.8% of 411 neonates were born with congenital CMV [6]. A study conducted in Indonesia revealed that 97.8% of 100 children aged 2–5 years with Autism Spectrum Disorder (ASD) were seropositive for CMV [7]. CMV frequently leads to latent and chronic infections, especially in paediatric populations and those with weakened immune systems [8]. Children with autism who are infected with CMV often exhibit compromised inflammatory and neuroinflammatory responses, potentially impacting brain development [9].

The association between CMV infection and autism remains underexplored. However, several studies have identified potential links. For instance, CMV infection during infancy, particularly before the age of 2 years, has been associated with an increased risk of epilepsy and ASD in childhood [10]. Another study reported that 3% of children with ASD and intellectual disabilities had congenital CMV infection, emphasising the importance of medical assessments and repeated hearing evaluations [11]. Similarly, a retrospective study in Nagasaki demonstrated a significantly higher prevalence of congenital CMV infection among children with ASD (7.4%) compared to the general population (0.31%) [12].

Other recent studies show that around 64 per 1000 children with congenital CMV receive an autism diagnosis, and they have a 2.5-times-higher likelihood of receiving an autism diagnosis [13]. CMV infection is known to cause increased inflammation by activating proinflammatory pathways that trigger the production of various proinflammatory cytokines, such as IL-1β, IL-6, TNF-α, and IL-8 [14]. CMV can be a lifelong infection and, in some conditions, can cause reactivation that triggers inflammation [15]. This condition triggers excessive immune system activation and damaging inflammation, increasing the risk of long-term complications such as developmental delays and neurological dysfunction [16]. CMV can affect behaviour through neurological effects, especially in children with congenital infection disturbances [17]. CMV can cause brain inflammation, affecting the central nervous system and causing symptoms such as developmental delays, behavioural problems, and sensory issues [18].

Behavioural disorders are common in children with autism, and their effects are substantial [19]. Symptoms related to different behavioural disorders frequently intersect with the fundamental traits of autism, leading to difficulties in diagnosis [20]. Autism Spectrum Disorder (ASD) is a multifaceted neurodevelopmental condition defined by impairments in social communication and the presence of restricted and repetitive behaviours and interests [21]. ASD is a multifactorial condition, with its aetiology involving both genetic and environmental factors [22]. Genetic underpinnings of ASD include single-nucleotide variants (SNVs), polygenic risk scores, and epigenetic modifications, all of which contribute to its complex pathophysiology [23] and potential interactions with environmental triggers such as CMV infections [24].

The behavioural development profile denotes the specific behavioural patterns that manifest in individuals over time, functioning as developmental milestones. Analysing the behavioural profile of children with autism is essential for identifying the characteristics of autism across different developmental stages [25].

The behavioural development profile of children with autism includes multiple dimensions, including sleep behaviour, eating behaviour, sensory profile, and social communication skills [26]. Children with autism often exhibit atypical eating behaviours, including significant food refusal, selective eating, or highly restricted dietary patterns [27]. Eating disturbances in children with autism are frequently associated with atypical sensory responses, including hyper-responsiveness (overreaction to stimuli), hypo-responsiveness (underreaction to stimuli that typically elicit a response), and sensation seeking (intense interest in sensory stimulation) [28].

Cytokine dysregulation denotes an imbalance in the synthesis of pro-inflammatory and anti-inflammatory cytokines. Research indicates that children with autism exhibit elevated levels of pro-inflammatory cytokines, including interleukin-6 (IL-6) and interleukin-1 beta (IL-1β), alongside reduced levels of anti-inflammatory cytokines, such as interleukin-10 (IL-10) [29]. This imbalance can result in chronic brain inflammation, referred to as neuroinflammation, which is associated with symptoms of ASD [30] Cytokine levels influence autistic behaviour in children with autism. Children with autism exhibit distinct cytokine profiles compared to neurotypical children [31]. Increased concentrations of proinflammatory cytokines, including IL-1β and IFN-γ, in children diagnosed with autism may result in persistent brain inflammation, which can impact neurological development and behaviour [32]. The cytokine profile in blood serves as a crucial biomarker for diagnosing and comprehending the pathophysiology of autism. Cytokines significantly influence autistic behaviour and may serve as a potential therapeutic approach for alleviating autism symptoms [33].

IL-6 and IL-1β are proinflammatory cytokines commonly linked to the pathophysiology of autism and may affect autistic behaviour [31]. Elevated levels of IL-6 and IL-1β play a crucial role in regulating immune responses and are frequently linked to chronic inflammation, which may affect brain development and behaviour in children with autism [30]. In children with autism, elevated levels of IL-1β are associated with cognitive dysfunction and deficits in social behaviour. Interventions aimed at reducing IL-1β activity have demonstrated the potential to alleviate specific symptoms associated with autism [33]. Multiple meta-analyses have investigated the involvement of IL-6 and IL-1β in autism, revealing that increased levels of these cytokines correlate with heightened autism symptom severity, especially in social communication and repetitive behaviours [32].

This study aimed to evaluate the association between CMV IgG levels and IL-6 and IL-1β cytokine profiles in children with autism aged 2–5 years to assess their correlation with autistic behaviours in the high-burden CMV region. Understanding this correlation is crucial, as it can significantly improve the management of autism in children within the intervention age range.

## 2. Materials and Methods

### 2.1. Study Design

This study is a cross-sectional study of 2–5-year-old children. The case group, defined as a group of children with Autism Spectrum Disorder (ASD), comprised 98 children (76 males and 22 females). The inclusion criteria were the following: (1) children aged 2–5 years; (2) with a diagnosis of ASD performed independently by a developmental paediatrician and a psychiatrist using the DSM-5 in combination with the CARS (Childhood Autism Rating Scale); (3) children without other organic diseases, hearing disorders, genetic metabolism diseases, mental and psychological disorders, and other neurodevelopmental disorders such as Attention-Deficit/Hyperactivity Disorder (ADHD) and Intellectual Disability (ID); and (4) children in stable medical conditions, not having an autoimmune disease.

The control group comprised 96 children without autism, which is mentioned in this study as children undergoing typical development (TD) (52 males and 44 females), who underwent examinations in the same period. The inclusion criteria were the following: (1) children aged 2–5 years; (2) conforming to the developmental process of children of the same age through routine examination and using the M-CHAT (Modified Checklist for Autism in Toddlers); (3) excluding other organic diseases, hearing disorders, neurodevelopmental disorders, and genetic metabolic diseases, as well as mental and psychological disorders; and (4) in stable medical conditions, not having an autoimmune disease. They were enrolled through Peduli Autism Spectrum Disorder (PASD), Jakarta, Indonesia, from 23 September to 23 December 2023. The Faculty of Medicine, University Indonesia Ethics Committee approved the study protocol, with reference number KET-824/UN2.F1/ETIK/PPM.00.02/2023. All of the participants’ parents gave written informed consent.

### 2.2. Assessment of Eating Behaviour and Sensory Profiles

The behavioural development profile in children with autism encompasses various aspects. This study used the BAMBI (Brief Autism Mealtime Behaviour Inventory) to assess eating behaviour and the SSP (Short Sensory Profile) to assess the sensory profile. The BAMBI was developed as the first standard to assess mealtimes and eating behaviours in children with autism. The scale initially consisted of 21 items, but the latest BAMBI has been reduced to 18 items determined by three factors: limited variety, food refusal, and autism characteristics [34]. The SSP is a shortened version of Dunn’s Sensory Profile caregiver questionnaire, initially developed as a screening tool to identify children with sensory processing difficulties. The SSP contains 38 items organised into seven subscales, including tactile sensitivity (7 items), taste/smell sensitivity (4 items), movement sensitivity (3 items), low responsiveness/sensation seeking (7 items), auditory filtering (6 items), low energy (6 items), and visual/auditory sensitivity (5 items) [35].

The validation and reliability testing of the SSP and BAMBI (Brief Autism Mealtime Behaviour Inventory) for the Indonesian context followed standard guidelines for adapting foreign instruments. The steps included the following: (1) Translation and Back-Translation: To ensure the accuracy of the translation, independent translators conducted a back-translation into English; (2) Cultural Adaptation: Items were reviewed by a panel of experts to ensure that the translated instruments were culturally appropriate and relevant for the Indonesian population; (3) Pilot Testing: The adapted instruments were piloted on a small sample of parents and caregivers to assess clarity, comprehension, and cultural relevance. Feedback from this process was incorporated to refine the instruments further; and (4) Validation and Reliability Testing: After finalising the translations, a larger sample was used to assess the psychometric properties of the instruments. Construct validity was evaluated using factor analysis, while reliability was assessed by calculating Cronbach’s alpha and test–retest reliability. The results confirmed that the SSP and BAMBI retained their validity and reliability after being adapted to the Indonesian context. These steps ensured that the instruments were scientifically robust and culturally appropriate for assessing sensory profiles and eating behaviours in Indonesian children with autism.

### 2.3. Sample Collection

EMLA (Eutectic Mixtures of Local Anaesthetics) cream was applied to the child’s hand area to induce local anaesthesia and reduce pain perception. For health screening and the scheduling of blood sampling, and to ensure accurate cytokine measurements, we implemented a rigorous health screening and scheduling protocol. Parents were required to complete a health questionnaire one week prior to the scheduled blood sampling, addressing their child’s current health status, recent symptoms (e.g., fever, other signs of illness), fluid intake, and history of recurrent conditions such as allergies. On the day of the blood sampling, paediatric residents and trained paediatric nurses conducted a thorough physical examination of the child to confirm their healthy condition. The blood sample was collected after ensuring that the child showed no signs of illness and was well hydrated.

A blood sample of approximately 3 mL was collected and transferred into an EDTA tube that contained an anticoagulant. The blood sample was centrifuged at room temperature for 10 min at 3500 rpm to obtain plasma and buffy coat. The plasma and buffy coat were stored at a temperature of −80 °C.

### 2.4. CMV Antibody Index Measurements

The antihuman IgG CMV was quantified from plasma samples at a 1:21 dilution utilising a Calbiotech CMV Antibody index kit (Calbiotech, El Cajon, CA, USA). The ELISA (Enzyme-Linked Immunosorbent Assay) test for detecting anti-CMV IgG required 100 microliters per sample. Incubation occurred for 20 min at room temperature before adding the enzyme conjugate. The wells underwent three wash cycles using 100 microliters of wash buffer. A 20-min incubation at room temperature was conducted before adding 100 microliters of TMB (Tetramethylbenzidine) substrate. A 10-min incubation at room temperature in the dark was subsequently conducted before adding 100 microliters of stop solution. Optical density was measured at a wavelength of 450 nm utilizing an ELISA Reader (Biotek, Santa Clara, CA, USA), as previously described [7].

### 2.5. IL-1β and IL-6 Measurements

The ELISA technique examines IL-1β (Komabiotech, Seoul, Republic of Korea) and IL-6 (Komabiotech, Seoul, Republic of Korea). The ELISA method was executed following the instructions supplied with each kit. The plasma was diluted at 1:30 for IL-1β and IL-6. The dilution factor was determined through an optimisation process using serial dilutions of 1:10, 1:30, 1:90, 1:270, 1:810, 1:2430, 1:7290, and 1:21870. The 1:30 dilution yielded the most optimal results, ensuring accurate and reliable cytokine detection. As no existing reference supported this specific dilution factor, the optimisation was conducted independently through experimental trials to establish the most suitable condition for this study. Plasma was added to ELISA plates previously coated with CMV antigen, IL-1β, and IL-6. Samples were added to the coated plates following incubation. The plates underwent a triple-washing process before the detecting antibody was applied. Streptavidin-HRP was added, followed by a signal amplifier, and subsequently washed. The TMB substrate was introduced and allowed to incubate for 10 min. Then, the stop solution was added when the incubation time had elapsed. Subsequently, the sample was analysed using an ELISA Reader (Biotek, Santa Clara, CA, USA) at 450 nm. Absorbance measurements were used to quantify IL-1β and IL-6 levels.

### 2.6. Statistical Analyses

Descriptive statistics on the characteristics of the experimental sample were presented separately for subjects with autism and those without autism. Data were presented as median with min–max values. The two groups were compared using the Mann–Whitney U Test for numerical data and the Chi-Square Test of Independence for categorical data. Correlation analysis was conducted using Spearman’s rank correlation test for numerical data. Data were analysed using SPSS version 22 and R Software 4-3.1 with the ggplot2, ggcorrplot, and ggpubr packages for data visualisation. Non-linear regression analysis used a generalised linear model and Stargazer package [36].

A normality test was performed on all data to assess the distribution. The results indicated that the data were not normally distributed. Therefore, non-parametric statistical methods were employed for further analysis. The sample size was determined using a power analysis to ensure sufficient statistical power to detect meaningful differences. The power analysis was based on an effect size, a significance level of 0.05, and a desired power of 0.80. Based on these parameters, this study included 98 participants in the ASD group and 96 participants in the TD group, ensuring the reliability of the findings.

## 3. Results

### 3.1. Demographic and Clinical Characteristics

There were significant demographic differences between children in the ASD and TD groups. The ASD group had a mean age of 4.19 years, compared to 3.55 years in the TD group (*p* < 0.001). Gender distribution also differed markedly, with a higher proportion of males in the ASD group (76 males vs. 22 females) compared to the TD group (55 males vs. 44 females), yielding a significant *p*-value of < 0.001. Although the age of the mothers was not significantly different between groups, fathers of children with ASD were significantly older (mean age 36.15 years) than the fathers of the children in the TD group (mean age 33.70 years) (Table 1).

The results showed that the two groups differed by gender and age, the mother’s and father’s age, eating behaviour, and sensory profile category using the BAMBI and SSP. The data demonstrate variation in these parameters between the ASD and TD groups. According to the eating behaviour parameters (using the BAMBI), 62% of children in the ASD group and 49% of children in the TD group had moderate issues in their eating behaviour. According to measurements obtained from the SSP, 53.1% of children in the ASD group exhibited moderate–severe issues in their sensory profiles. Conversely, 64.6% of children in the TD group exhibited no issues in their sensory profile. This establishes the distinction in sensory profiles between the two groups (Table 1).

### 3.2. Comparative Analysis of ASD and TD Groups

**Figure 1:**
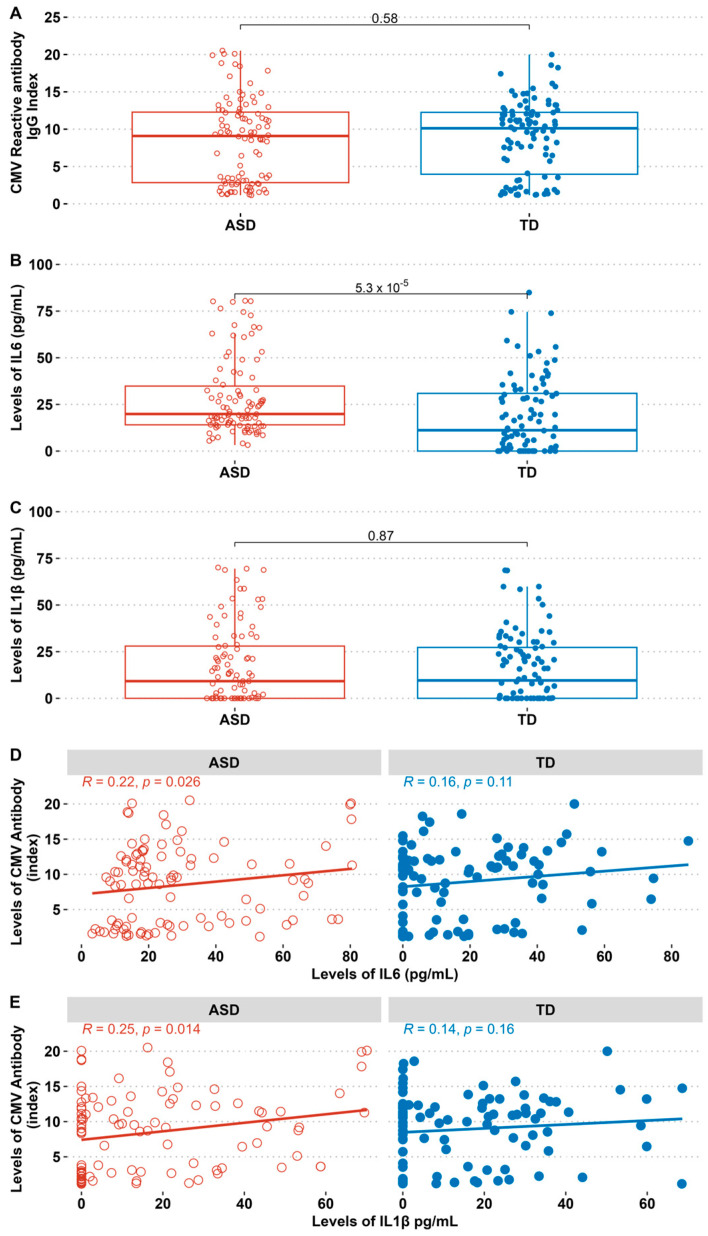
Differences in CMV-reactive antibody levels, IL-6, and IL-1β between the ASD and TD groups and correlations of CMV-reactive antibody with IL-6 and IL-1β levels: (**A**) comparison of CMV-reactive antibody levels between the ASD and TD groups, (**B**) IL-6 levels in the ASD and TD groups, (**C**) IL-1β levels in the ASD and TD groups, (**D**) correlation between CMV-reactive antibody levels and IL-6 levels, and (**E**) correlation between CMV-reactive antibody levels and IL-1β levels. Data are presented as median with interquartile range (IQR). ASD = Autism Spectrum Disorder, TD = Typically Developing. Comparison analysis using the Mann–Whitney Test (**A**–**C**). The Spearman Rank correlation test calculated the correlation coefficient (R) and *p*-value (**D**,**E**).

In the comparative analysis between the ASD and TD groups, the CMV-reactive IgG antibody index did not show a significant difference (Figure 1A). IL-6 levels of the ASD group were significantly different and higher than those in the TD group (Figure 1B). IL-1β concentrations did not differ significantly between the two groups (Figure 1C).

### 3.3. Correlation Analysis of IL-6 and IL-1β with CMV, Eating Behaviours, and Sensory Profiles

**Figure 2:**
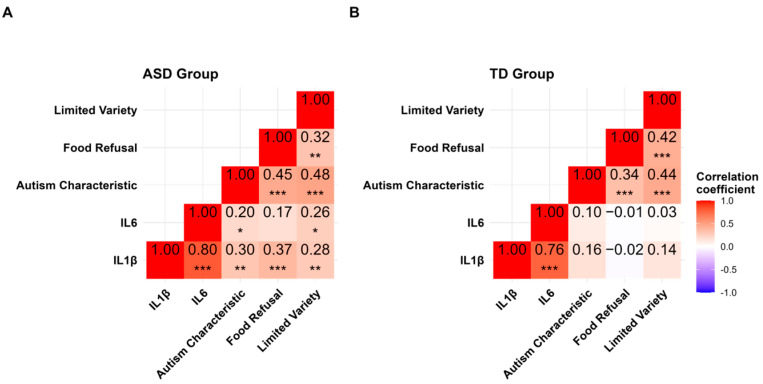
Correlation analysis of eating behaviours with IL-6 and IL-1β: (**A**) ASD and (**B**) TD groups. ASD = Autism Spectrum Disorder, TD = Typically Developing. Statistical significance is indicated as follows: * *p* < 0.05, ** *p* < 0.01, and *** *p* < 0.001 using Spearman’s correlation test.

**Figure 3:**
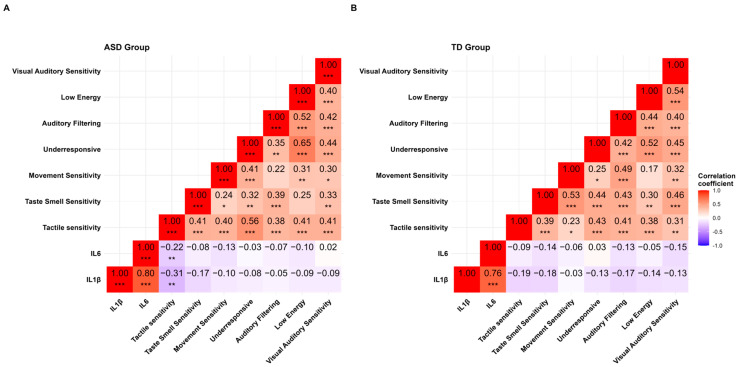
Correlation analysis of sensory profiles with IL-6 and IL-1β: (**A**) ASD and (**B**) TD groups. ASD = Autism Spectrum Disorder, TD = Typically Developing. Statistical significance is indicated as follows: * *p* < 0.05, ** *p* < 0.01, and *** *p* < 0.001 using Spearman’s correlation test.

The levels of CMV antibodies showed a positive correlation with IL-1β and IL-6 in the ASD group (Figure 1D,E). Notably, IL-6 was directly linked to CMV IgG and positively correlated with eating behaviour in the ASD group but not in the TD group (Figure 2). The data might emphasise the role of CMV in fostering proinflammation that could impact children’s development. There was a correlation between IL-6 and IL-1β and tactile sensitivity (sub-scale of the sensory profiles) in the ASD group only (Figure 3). The IL-1β positively correlated with eating behaviour (Figure 2) and negatively correlated with sensory profiles. The SSP-38 item has a reversed interpretation; higher scores indicate fewer sensory issues. Therefore, lower scores in tactile sensitivity (indicating sensory difficulties) are associated with higher IL-6 and IL-1β (Figure 3).

The correlation analysis of BAMBI sub-scales (18 items) with IL-6 and I IL-1β is presented in Figure 2. The results show that, within the ASD group, the subscales of food refusal, autism characteristics, and limited variety display a correlation with IL-6 and IL-1β. There are no significant correlations in the TD group.

The correlation analysis between the SSP sub-scales (38 items) and IL-6 and IL-1β was observed only for the tactile sensitivity sub-scale within the ASD group. In the TD group, correlations were identified between tactile sensitivity, taste/smell sensitivity, and IL-1β levels (Figure 3). Multi-variable non-linear regressions were conducted to assess age and gender as confounding factors in ASD, with groups detailed in thesupplementary data. Food refusal (Supplementary Tables S1 and S2), characteristics of autism (Supplementary Tables S3 and S4), limited variety (Supplementary Tables S5 and S6), and tactile sensitivity (Supplementary Tables S7 and S8) served as dependent variables in relation to IL-1β, IL-6, age, and gender. Model 1 incorporated IL-1β, IL-6, age, and gender, whereas Model 2 excluded IL-1β, and Model 3 excluded IL-6. Age and gender were the sole confounding factors identified in the TD group, as shown in Supplementary Table S2 for food refusal and Supplementary Table S6 for a limited variety of variables. IL-1β and IL-6 exhibited multicollinearity as indicated by the Variance Inflation Factor (VIF). Thus, Models 2 and 3 provided the best fit for the data that showed an estimated value of IL-1β and IL-6 to the dependent variables.

## 4. Discussion

Children with autism commonly experience challenges in eating behaviours and sensory profiles. In this study, we observed that among 98 children with ASD, 63.3% demonstrated severe issues in eating behaviour and 53.1% demonstrated issues in sensory profiles. These findings align with previous studies indicating that children with ASD exhibit distinct and often more problematic eating behaviours compared to TD children, particularly regarding food refusal and limited dietary variety (*p* < 0.001) [37]. In this study, we also identified that food refusal and limited variety, markers of eating behaviour, were strongly correlated with IL-6 and IL-1β levels in the ASD group.

The IL-6 levels in this study show a significant correlation with the CMV IgG antibody index in the ASD group. This suggests that CMV’s influence on IL-6 cytokine activity occurs exclusively in children with autism. CMV infection can trigger a robust immune response, with CMV-specific IgG antibodies in children possibly indicating prior infection that may contribute to pro-inflammatory changes in the central nervous system, increasing pro-inflammatory cytokines such as IL-6, which can impact behaviour and development [38]. CMV stimulates pro-inflammatory cytokine production through the activation of multiple signalling pathways, including NF-κB and MAPK pathways [39]. The elevation of IL-6 is associated with the body’s response to CMV infection, through the regulation of T cells and NK cells [40]. Additionally, IL-1β plays a role in CMV-induced inflammation by activating more macrophages and dendritic cells [41].

Several studies also indicate that increased levels of IL-6 and IL-1β induced by CMV infection may impact the nervous system, particularly in patients with neurological disorders such as autism or other neurodegenerative diseases [42]. Cytokines can affect neural development and behaviour by disrupting microglial and astrocyte activity in the brain [43]. CMV is also suspected to induce epigenetic changes within host cells, resulting in prolonged IL-6 and IL-1β production, creating chronic inflammation. This implies that even latent CMV infection may induce low-level, continuous IL-6 and IL-1β expression, potentially impacting the immune system and leading to long-term immune dysfunction [8].

Previous studies have shown that children with ASD often exhibit elevated levels of these cytokines, which may be associated with various behavioural symptoms, including those related to eating behaviour [44]. IL-6 and IL-1β are pro-inflammatory cytokines crucial in the immune response [45]. Research by Gones and Ashwood also highlights that cytokine dysregulation, involving IL-6 and IL-1β, can influence both behaviour and physiology, impacting eating behaviours in children with ASD. This dysregulation may lead to increased sensitivity to certain foods or highly restricted food preferences [46].

In our research, we explored the relationship between IL-6, IL-1β, and eating behaviour in TD children, which is less prevalent than in children with ASD. IL-6 and IL-1β can influence appetite and eating behaviour through systemic inflammation and cytokine-induced anorexigenic (appetite-reducing) effects, particularly during acute inflammatory states [47]. The effects of cytokines may also impact the sensory profiles of children with ASD. Sensory profiles reflect how individuals perceive, respond to, and process sensory information from their surroundings [48]. Another study identified significant differences in sensory profiles among children and adolescents with autism, finding that distinct sensory responses can affect daily functioning [49]. Among children with ASD, tactile sensitivity is the most frequently affected aspect.

Our study observed that tactile sensitivity, part of the SSP-38 item subscale, correlated strongly with IL-6 and IL-1β exclusively in the ASD group. Another study examining sensory subtypes in children with autism through the SSP identified a distinct sensory profile for children with ASD. They concluded that sensory processing disorders are a core feature of ASD rather than a secondary characteristic [50]. Sensory response differences in children with autism typically manifest as either hypersensitivity or hyposensitivity. Sensory stimuli such as sound, light, touch, taste, and movement can be particularly disruptive [51]. Studies utilising the SSP frequently find that not all children with autism exhibit identical sensory processing patterns. Some children may display a mix of hypersensitivity and hyposensitivity depending on the type of stimulus or specific context [28]. These sensory processing variations in children with autism suggest the need for more individualised interventions and support [52].

In this study, IL-6 and IL-1β strongly correlated with eating behaviour and sensory profiles in the ASD group. Notably, IL-6 levels were higher in the ASD group than in the TD group. Immune biomarker profiles suggest that children with ASD may have distinct IL-6 levels, differentiating them from TD children [46]. The dysregulation of cytokine profiles in children with ASD points to excessive immune activation, which could affect immune system quality [53].

Our findings indicate that IL-6 concentrations were significantly higher in the ASD group compared to the TD group (*p* < 0.001). This finding aligns with research by Ashwood et al., which also observed elevated IL-6 levels in children with ASD [54]. A study on children with ASD in China supports these findings, identifying IL-6 as a factor influencing autism risk and symptom severity, although significantly elevated levels did not impact autism risk and symptom severity [55]. Consistently high IL-6 levels in children with ASD suggest underlying systemic inflammation and neuroinflammation [56].

Thus, IL-6 may contribute to autism pathogenesis through neuroinflammatory mechanisms. IL-6 can induce brain inflammation, potentially disrupting neural development and synaptic function [57]. This hypothesis is reinforced by findings of elevated IL-6 in the cerebrospinal fluid and brain tissue of children with ASD, suggesting that such inflammation may alter neural connectivity and influence brain functions associated with behaviour and communication [58]. IL-6 also impacts neurotransmitter systems like dopamine and serotonin, which have been linked to behaviour and clinical symptoms in autism [59]. While some studies suggest a relationship between peripheral cytokine profiles and autism, the evidence remains limited, and there is no clear consensus on using cytokine profiles as a diagnostic biomarker for ASD [32]. Further research is needed to confirm these findings and determine their clinical relevance.

The limitation of this study is the lack of assessment for CMV IgM antibodies and quantitative PCR, which would have clarified the distinction between active and past CMV infections. Additionally, the 2–5-year age group, which includes Typically Developing (TD) children experiencing normal feeding challenges, complicates the interpretation of eating behaviour parameters, as age-related feeding issues may confound results and comparisons with the ASD group.

To address these limitations, future studies should assess CMV IgM antibodies and perform quantitative PCR to differentiate between active and past infections. Additionally, broader age ranges and longitudinal designs are needed to track the long-term effects of CMV on neurodevelopmental outcomes in both children with ASD and TD children. This could reveal how infection impacts development and its persistence across stages. Controlling for feeding behaviours in TD children would also help reduce confounding factors and improve result interpretation. Moreover, to strengthen the findings, additional experiments measuring IL-6 and IL-1β levels in cerebrospinal fluid (CSF) would be valuable, as blood levels alone may not fully reflect brain-specific processes. This would provide a more comprehensive understanding of the neuroinflammatory impact of CMV on neurodevelopment.

## 5. Conclusions

There is a high proportion of moderate–severe issues with sensory profiles and eating behaviour in children with ASD compared with TD children. We found high levels of IL-6 were observed in an ASD group in contrast to a TD group. Food refusal, limited variety, and tactile sensitivity are linked to IL1β and IL-6. Future studies should measure IL-6 and IL-1β in cerebrospinal fluid, as blood levels may not fully reflect brain-specific processes. Additionally, tightening inclusion criteria and using more comprehensive instruments will enhance the understanding of the correlation between cytokine biomarkers and autistic behaviours.

## Figures and Tables

**Table 1:** Demographic and clinical characteristics of the subject.

Characteristics	ASD (*n* = 98)	TD (*n* = 96)	
	Median (Min-Max)	Median (Min-Max)	*p*-Values
Children Gender (M:F)	76:22	55:44	**<0.001** ^a^
Children Age (years)	4.3 (2.4–5.0)	3.4 (2.0–5.0)	**<0.001** ^b^
Mother Age (years)	32 (25–45)	32 (26–43)	0.059 ^b^
Father Age (years)	35 (27–60)	33 (27–46)	**0.006** ^b^
	* n * (%)	* n * (%)	
BAMBI—18 item			
Mild issues	20 (20.4)	42 (43.8)	
Moderate issues	62 (63.3)	49 (51.0)	** <0.001 ** ^a^
Severe issues	16 (16.3)	5 (5.2)	
SSP—38 item			
No issues	18 (18.4)	62 (64.6)	
Mild issues	28 (28.6)	23 (24.0)	** <0.001 ** ^a^
Moderate to severe issues	52 (53.1)	11 (11.5)	

Bold: *p*-value ≤ 0.05 (comparative analysis); ^a^ Chi-Square Test of Independence; ^b^ Mann–Whitney U Test; BAMBI = Brief Autism Mealtime Behaviour Inventory; SSP = Short Sensory Profile.

## Data Availability

The data supporting the findings of this study are available upon reasonable request from the corresponding author. Data are not publicly available due to ethical restrictions related to participant confidentiality.

## Supplementary Materials

The following supporting information can be downloaded at: https://www.mdpi.com/article/10.3390/biomedicines13020338/s1, Supplementary Table S1: Multiple regression model for food refusal of children with autism; Supplementary Table S2: Multiple regression models for food refusal of typical development children; Supplementary Table S3: Multiple regression models for autism characteristics of children with autism; Supplementary Table S4: Multiple regression models for autism characteristic of typical development children; Supplementary Table S5: Multiple regression models for limited variety of children with autism; Supplementary Table S6: Multiple regression models for a limited variety of typical development children; Supplementary Table S7: Multiple regression models for tactile sensitivity of children with autism; Supplementary Table S8: Multiple regression models for tactile sensitivity of typical development children.
