# Supplementary material for: Cytomegalovirus-Reactive IgG Correlates with Increased IL-6 and IL-1β Levels, Affecting Eating Behaviours and Tactile Sensitivity in Children with Autism
Source: Biomedicines. 2025 Feb 2;13(2):338. doi: 10.3390/biomedicines13020338 (PMC11852405; doi:10.3390/biomedicines13020338)
Supplement: Supplementary file 1 [file biomedicines-13-00338-s001.zip › Supplementary Table S1.pdf]

Supplementary Table S1. Multiple regression model for food refusal of children with autism

	Dependent variable:		
	Food Refusal		
	(1)	(2)	(3)
CMV IgG	-0.07	-0.07	-0.05
	(0.07)	(0.07)	(0.07)
IL1B	0.08*	0.05**	
	(0.04)	(0.02)	
IL6	-0.03		0.04*
	(0.04)		(0.02)
Age	0.08	0.03	-0.03
	(0.51)	(0.50)	(0.52)
Gender	-0.10	-0.09	-0.06
	(0.90)	(0.89)	(0.91)
Constant	9.99***	9.76***	9.59***
	(2.43)	(2.40)	(2.46)
Observations	98	98	98
Log Likelihood	-261.82	-262.16	-264.20
Akaike Inf. Crit.	535.63	534.33	538.41

Note: *p<0.05; **p<0.01; ***p<0.001
