# Supplementary material for: Cytomegalovirus-Reactive IgG Correlates with Increased IL-6 and IL-1β Levels, Affecting Eating Behaviours and Tactile Sensitivity in Children with Autism
Source: Biomedicines. 2025 Feb 2;13(2):338. doi: 10.3390/biomedicines13020338 (PMC11852405; doi:10.3390/biomedicines13020338)
Supplement: Supplementary file 1 [file biomedicines-13-00338-s001.zip › Supplementary Table S2.pdf]

**Supplementary Table S2. Multiple regression models for food refusal of typical development children**

| | *Dependent variable:* | | |
|---|---|---|---|
| | Food Refusal | | |
| | (1) | (2) | (3) |
| CMV IgG | -0.02 | -0.02 | -0.02 |
| | (0.06) | (0.06) | (0.06) |
| IL6 | -0.01 | | 0.02 |
| | (0.03) | | (0.02) |
| Age | -0.87* | -0.84* | -0.80* |
| | (0.33) | (0.33) | (0.33) |
| Gender | 0.83 | 0.72 | 0.56 |
| | (0.64) | (0.60) | (0.62) |
| Constant | 10.77*** | 10.77*** | 11.02*** |
| | (1.51) | (1.51) | (1.52) |
| Observations | 96 | 96 | 96 |
| Log Likelihood | -237.08 | -237.23 | -238.40 |
| Akaike Inf. Crit. | 486.15 | 484.46 | 486.80 |

*Note:* *p<0.05; **p<0.01; ***p<0.001
