# Supplementary material for: Cytomegalovirus-Reactive IgG Correlates with Increased IL-6 and IL-1β Levels, Affecting Eating Behaviours and Tactile Sensitivity in Children with Autism
Source: Biomedicines. 2025 Feb 2;13(2):338. doi: 10.3390/biomedicines13020338 (PMC11852405; doi:10.3390/biomedicines13020338)
Supplement: Supplementary file 1 [file biomedicines-13-00338-s001.zip › Supplementary Table S3.pdf]

Supplementary Table S3. Multiple regression models for autism characteristics of children with autism

	Dependent variable:		
	Autism Characteristic		
	(1)	(2)	(3)
CMV IgG	0.03	0.04	0.06
	(0.05)	(0.05)	(0.06)
IL1B	0.09**	0.05**	
	(0.03)	(0.01)	
IL6	-0.05		0.03
	(0.03)		(0.01)
Age	-0.30	-0.39	-0.43
	(0.40)	(0.40)	(0.42)
Gender	0.61	0.64	0.66
	(0.71)	(0.71)	(0.74)
Constant	11.27***	10.88***	10.81***
	(1.92)	(1.92)	(2.00)
Observations	98	98	98
Log Likelihood	-238.68	-240.27	-243.58
Akaike Inf. Crit.	489.36	490.55	497.15

Note: *p<0.05; **p<0.01; ***p<0.001
