# Supplementary material for: Cytomegalovirus-Reactive IgG Correlates with Increased IL-6 and IL-1β Levels, Affecting Eating Behaviours and Tactile Sensitivity in Children with Autism
Source: Biomedicines. 2025 Feb 2;13(2):338. doi: 10.3390/biomedicines13020338 (PMC11852405; doi:10.3390/biomedicines13020338)
Supplement: Supplementary file 1 [file biomedicines-13-00338-s001.zip › Supplementary Table S4.pdf]

**Supplementary Table S4. Multiple regression models for autism characteristic of typical development children**

| | *Dependent variable:* | | |
|---|---|---|---|
| | Autism Characteristic | | |
| | (1) | (2) | (3) |
| CMV IgG | 0.002 | -0.001 | 0.001 |
| | (0.04) | (0.04) | (0.04) |
| IL1B | 0.02 | 0.01 | |
| | (0.02) | (0.01) | |
| IL6 | -0.01 | | 0.0002 |
| | (0.02) | | (0.01) |
| Age | -0.19 | -0.16 | -0.16 |
| | (0.23) | (0.23) | (0.23) |
| Gender | $0.90^{*}$ | 0.81 | 0.80 |
| | (0.45) | (0.43) | (0.43) |
| Constant | $9.18^{***}$ | $9.18^{***}$ | $9.27^{***}$ |
| | (1.07) | (1.07) | (1.06) |
| Observations | 96 | 96 | 96 |
| Log Likelihood | -203.66 | -203.89 | -204.01 |
| Akaike Inf. Crit. | 419.31 | 417.79 | 418.02 |

*Note: *p<0.05; **p<0.01; ***p<0.001*
