# Supplementary material for: Cytomegalovirus-Reactive IgG Correlates with Increased IL-6 and IL-1β Levels, Affecting Eating Behaviours and Tactile Sensitivity in Children with Autism
Source: Biomedicines. 2025 Feb 2;13(2):338. doi: 10.3390/biomedicines13020338 (PMC11852405; doi:10.3390/biomedicines13020338)
Supplement: Supplementary file 1 [file biomedicines-13-00338-s001.zip › Supplementary Table S5.pdf]

Supplementary Table S5. Multiple regression models for limited variety of children with autism

	Dependent variable:		
	Limited Variety		
	(1)	(2)	(3)
CMV_IgG	0.02	0.02	0.03
	(0.08)	(0.08)	(0.08)
IL1B	0.06	0.05**	
	(0.04)	(0.02)	
IL6	-0.001		0.05*
	(0.04)		(0.02)
Age	0.48	0.47	0.40
	(0.58)	(0.58)	(0.58)
Gender	-0.37	-0.37	-0.34
	(1.03)	(1.02)	(1.03)
Constant	13.71***	13.70***	13.43***
	(2.78)	(2.75)	(2.78)
Observations	98	98	98
Log Likelihood	-275.20	-275.20	-276.05
Akaike Inf. Crit.	562.40	560.40	562.11

*Note:*p<0.05; **p<0.01; ***p<0.001*
