# Supplementary material for: Cytomegalovirus-Reactive IgG Correlates with Increased IL-6 and IL-1β Levels, Affecting Eating Behaviours and Tactile Sensitivity in Children with Autism
Source: Biomedicines. 2025 Feb 2;13(2):338. doi: 10.3390/biomedicines13020338 (PMC11852405; doi:10.3390/biomedicines13020338)
Supplement: Supplementary file 1 [file biomedicines-13-00338-s001.zip › Supplementary Table S6.pdf]

Supplementary Table S6. Multiple regression models for a limited variety of typical development children

	Dependent variable:		
	Limited Variety		
	(1)	(2)	(3)
CMV IgG	-0.05	-0.06	-0.05
	(0.07)	(0.07)	(0.07)
IL1B	0.09^{**}	0.04^{*}	
	(0.03)	(0.02)	
IL6	-0.05		0.01
	(0.03)		(0.02)
Age	0.73^{*}	0.84^{*}	0.85^{*}
	(0.36)	(0.35)	(0.37)
Gender	2.11^{**}	1.72^{*}	1.60^{*}
	(0.68)	(0.66)	(0.68)
Constant	7.34^{***}	7.37^{***}	7.81^{***}
	(1.62)	(1.64)	(1.67)
Observations	96	96	96
Log Likelihood	-243.47	-245.27	-247.46
Akaike Inf. Crit.	498.93	500.53	504.92

*Note:**p<0.05; **p<0.01; ***p<0.001*
