# Supplementary material for: Cytomegalovirus-Reactive IgG Correlates with Increased IL-6 and IL-1β Levels, Affecting Eating Behaviours and Tactile Sensitivity in Children with Autism
Source: Biomedicines. 2025 Feb 2;13(2):338. doi: 10.3390/biomedicines13020338 (PMC11852405; doi:10.3390/biomedicines13020338)
Supplement: Supplementary file 1 [file biomedicines-13-00338-s001.zip › Supplementary Table S7.pdf]

Supplementary Table S7. Multiple regression models for tactile sensitivity of children with autism

	Dependent variable:		
	Tactile Sensitivity		
	(1)	(2)	(3)
CMV IgG	0.06	0.06	0.02
	(0.13)	(0.13)	(0.13)
IL1B	-0.14	-0.11**	
	(0.07)	(0.04)	
IL6	0.03		-0.09*
	(0.07)		(0.04)
Age	-0.95	-0.90	-0.76
	(1.00)	(0.98)	(1.00)
Gender	1.04	1.03	0.97
	(1.75)	(1.74)	(1.77)
Constant	40.81***	41.01***	41.48***
	(4.73)	(4.68)	(4.78)
Observations	98	98	98
Log Likelihood	-327.34	-327.41	-329.13
Akaike Inf. Crit.	666.67	664.82	668.26

*Note: *p<0.05; **p<0.01; ***p<0.001*
