# Supplementary material for: Cytomegalovirus-Reactive IgG Correlates with Increased IL-6 and IL-1β Levels, Affecting Eating Behaviours and Tactile Sensitivity in Children with Autism
Source: Biomedicines. 2025 Feb 2;13(2):338. doi: 10.3390/biomedicines13020338 (PMC11852405; doi:10.3390/biomedicines13020338)
Supplement: Supplementary file 1 [file biomedicines-13-00338-s001.zip › Supplementary Table S8.pdf]

Supplementary Table S8. Multiple regression models for tactile sensitivity of typical development children

	Dependent variable:		
	Tactile Sensitivity		
	(1)	(2)	(3)
CMV IgG	0.15	0.16	0.15
	(0.10)	(0.10)	(0.10)
IL1B	-0.09	-0.07*	
	(0.05)	(0.03)	
IL6	0.02		-0.05
	(0.05)		(0.03)
Age	0.15	0.11	0.02
	(0.55)	(0.54)	(0.55)
Gender	-0.65	-0.52	-0.16
	(1.05)	(0.99)	(1.02)
Constant	42.68***	42.67***	42.22***
	(2.49)	(2.48)	(2.51)
Observations	96	96	96
Log Likelihood	-284.84	-284.93	-286.53
Akaike Inf. Crit.	581.67	579.86	583.05

*Note:*p<0.05; **p<0.01; ***p<0.001*
